# Supplementary material for: IODVA1, a guanidinobenzimidazole derivative, targets Rac activity and Ras-driven cancer models
Source: PLoS One. 2020 Mar 12;15(3):e0229801. doi: 10.1371/journal.pone.0229801 (PMC7067412; doi:10.1371/journal.pone.0229801)
Supplement: S1 Raw images — (PDF) [file pone.0229801.s001.pdf]

2A

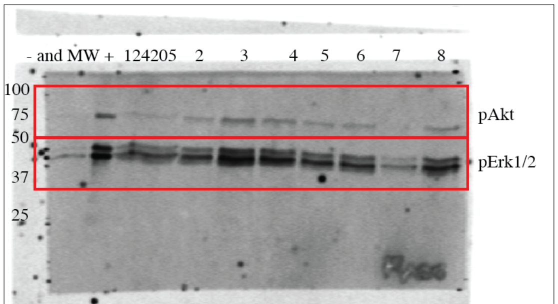

outlined in red belongs to panel 2A pAkt (top) and pErk1/2 (bottom)
captured with anti-rabbit Eu ab using SpectraMax i3 ScanLater

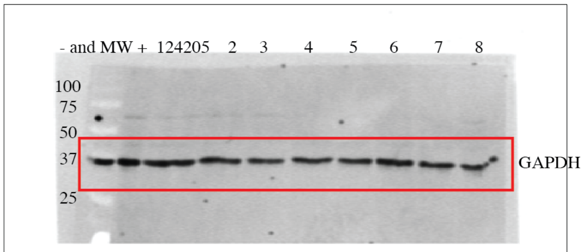

captured with anti-mouse Eu ab using SpectraMax i3 ScanLater
outlined in red belongs to panel 2A GAPDH

2B

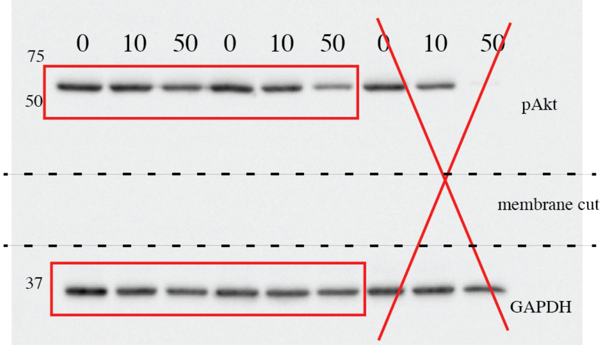

captured with anti-rabbit HRP ab using Bio-Rad chemiluminescent system
outlined in red belongs to panel 2B pAkt (upper piece) and GAPDH (lower piece)

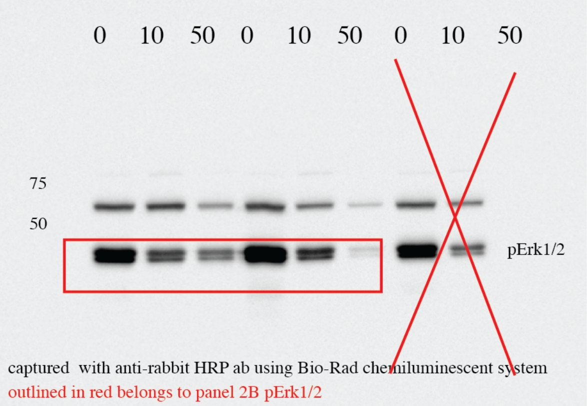

captured with anti-rabbit HRP ab using Bio-Rad chemiluminescent system
outlined in red belongs to panel 2B pErk1/2

Expression of HRas in transduced 3T3 cells for part of panel 2C was determined in early 2014 when the cell lines were established and orginal membranes could not be located

2C

pERK1/2

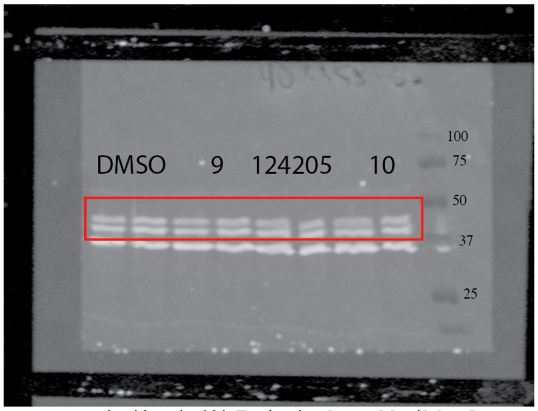

captured with anti-rabbit Eu ab using SpectraMax i3 ScanLater
outlined in red belongs to panel 2C pErk1/2, 10 uM treatment

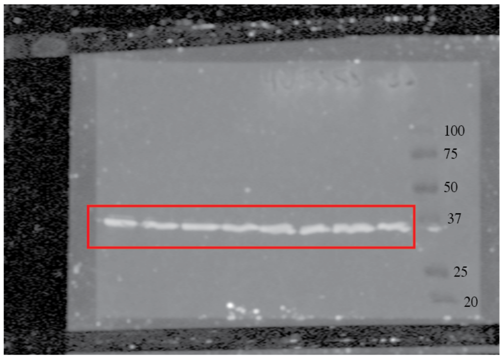

GAPDH

captured with anti-mouse Eu ab using SpectraMax i3 ScanLater
outlined in red belongs to panel 2C GAPDH, 10 uM treatment

75
50
pERK1/2 37
25

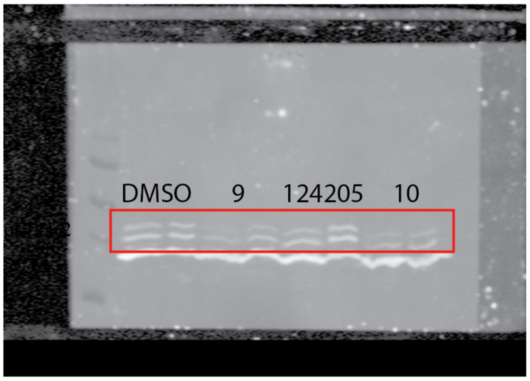

captured with anti-rabbit Eu ab using SpectraMax i3 ScanLater
outlined in red belongs to panel 2C pErk1/2, 50 uM treatment

75
50
37
25

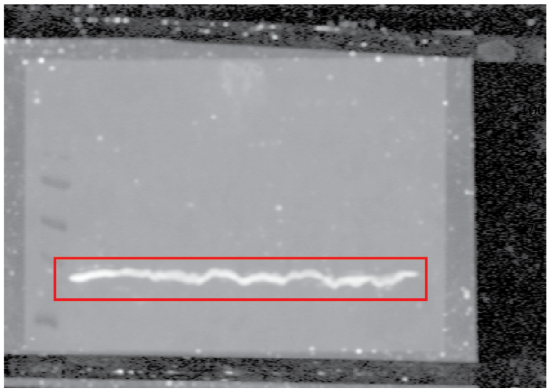

GAPDH

captured with anti-mouse Eu ab using SpectraMax i3 ScanLater
outlined in red belongs to panel 2C GAPDH, 50 uM treatment

4C

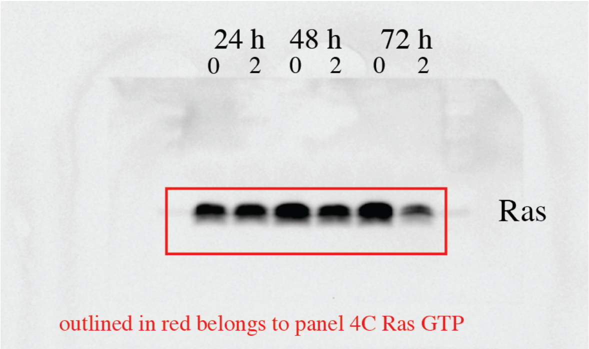

captured with anti-mouse HRP using Bio-Rad chemiluminescent system
Clarity Max ECL substrate

With molecular weight markers overlayed

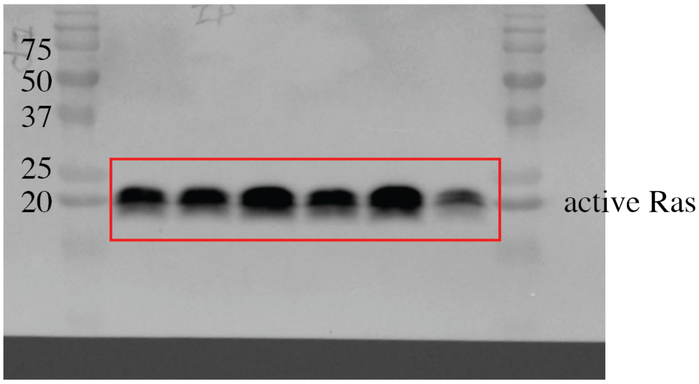

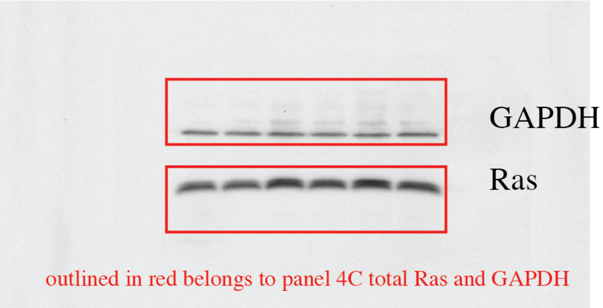

captured with anti-mouse HRP using Bio-Rad chemiluminescent system
Clarity Max ECL substrate

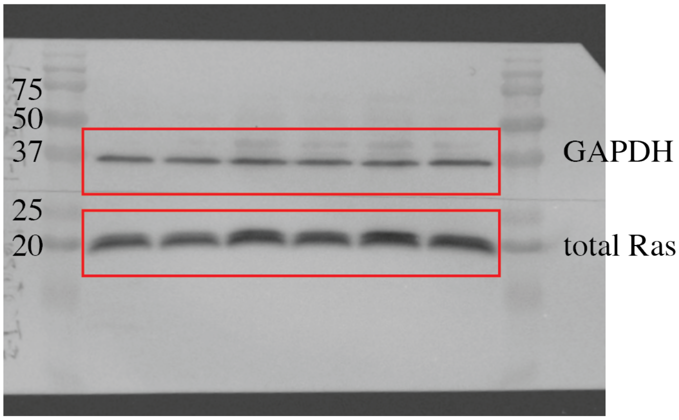

5C

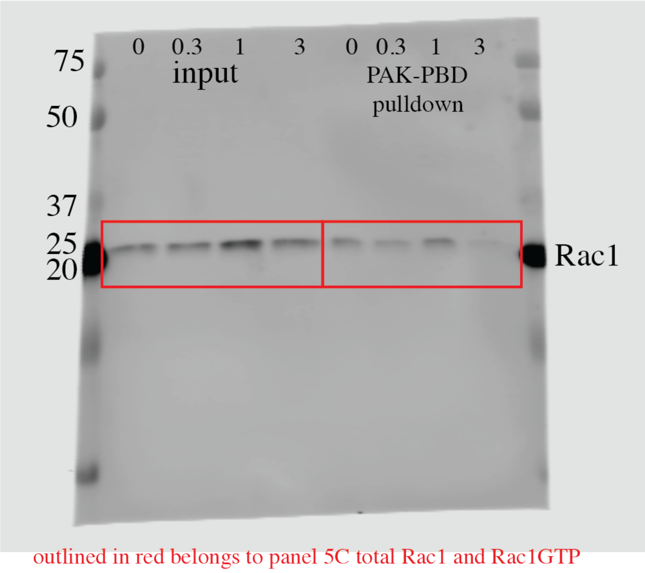

outlined in red belongs to panel 5C total Rac1 and Rac1GTP

captured with anti-mouse HRP using Bio-Rad chemiluminescent system
Clarity Max ECL substrate

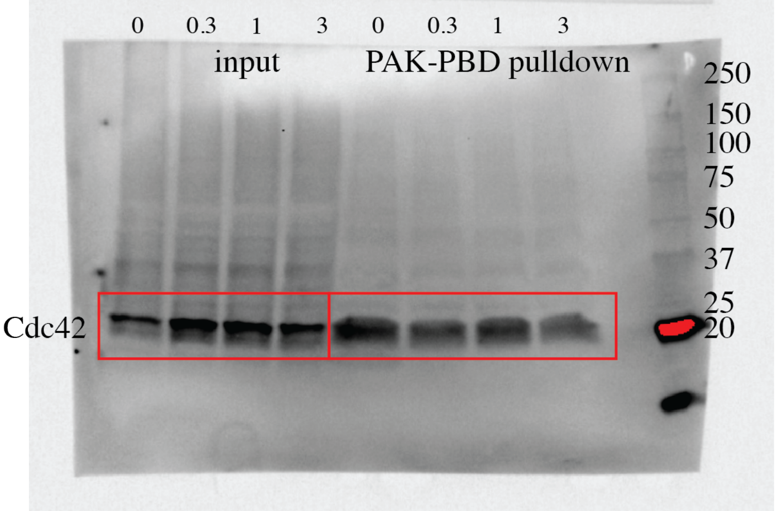

outlined in red belongs to panel 5C total Cdc42 and Cdc42GTP

captured with anti-rabbit HRP using Bio-Rad chemiluminescent system
Clarity Max ECL substrate

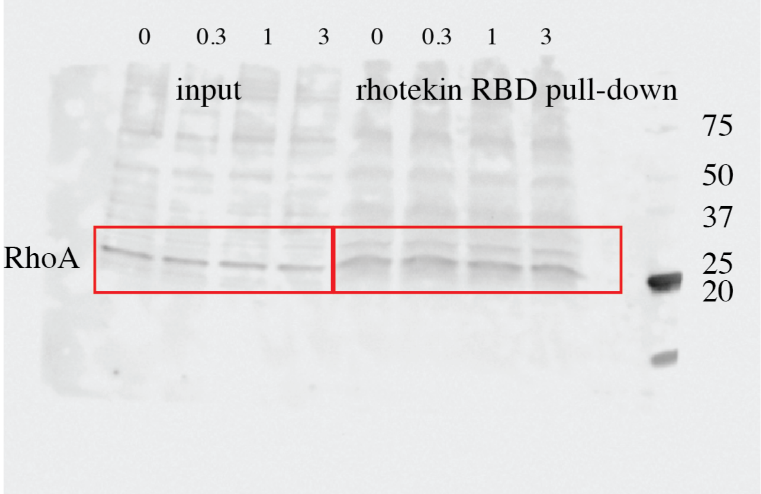

outlined in red belongs to panel 5C total RhoA and RhoAGTP

captured with anti-mouse HRP using Bio-Rad chemiluminescent system
SuperSignal West Pico Substrate

5D

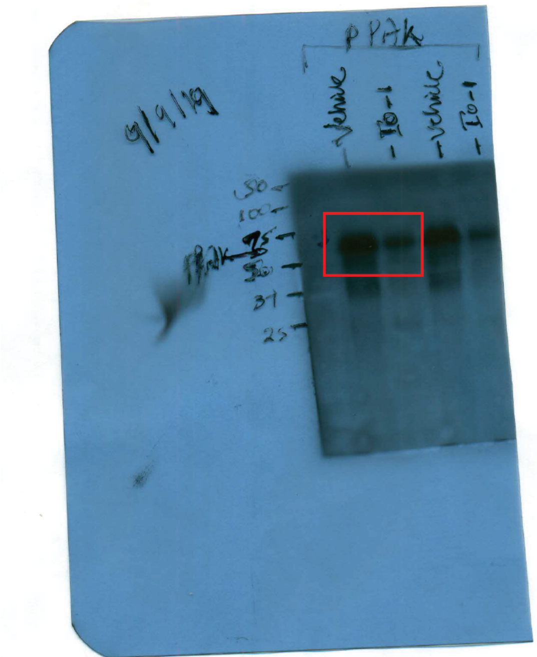

outlined in red belongs to panel 5 pPak1

captured with anti-rabbit HRP using traditional ECL/ X-Ray film

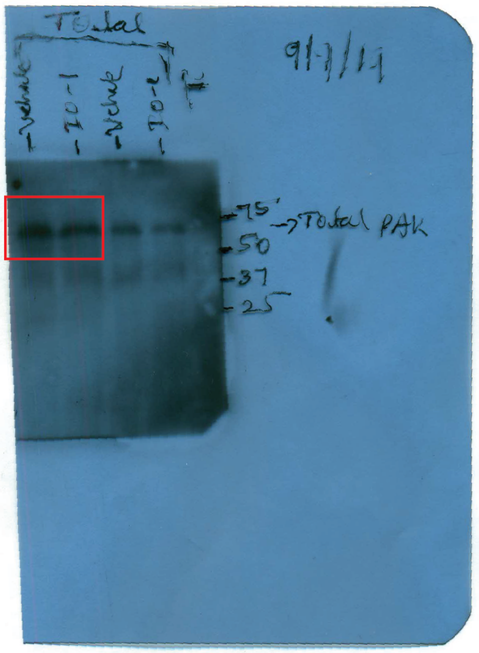

outlined in red belongs to panel 5 t-Pak

captured with anti-rabbit HRP using traditional ECL/ X-Ray film

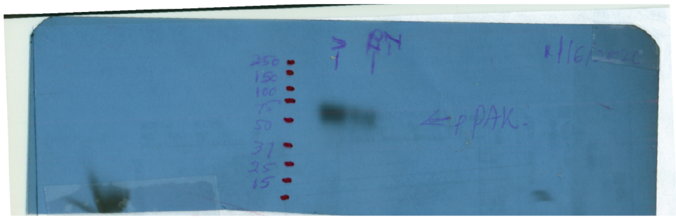

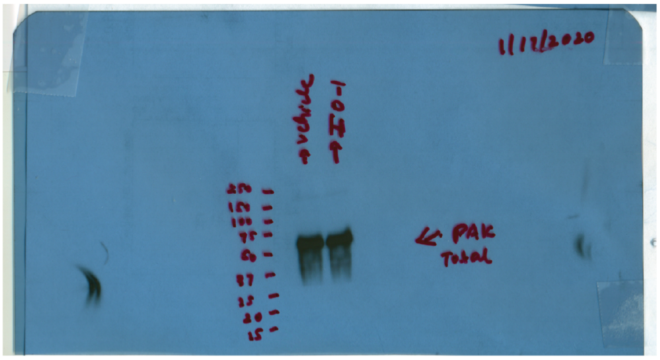

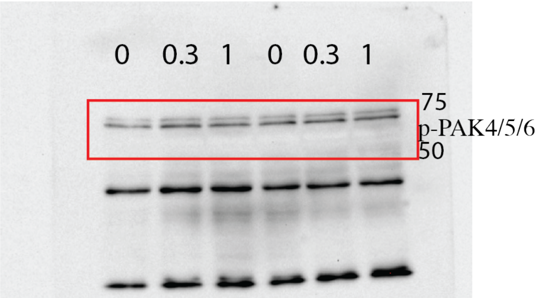

outlined in red belongs to panel 5D p-Pak4/5/6

captured with anti-rabbit HRP using Bio-Rad chemiluminescent system SuperSignal West Pico

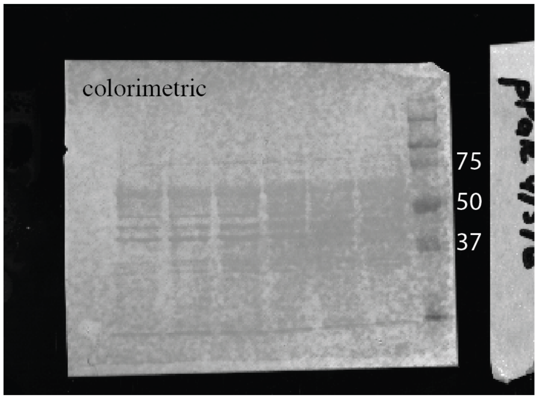

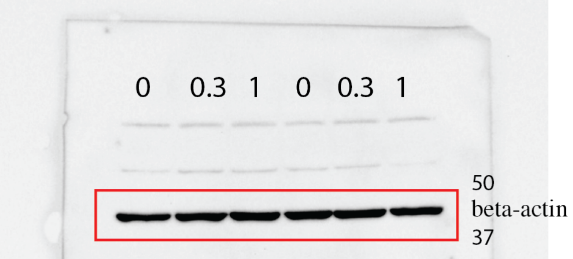

outlined in red belongs to panel 5D b-actin

captured with anti-mouse HRP using Bio-Rad chemiluminescent system Clarity MAX ECL reagent

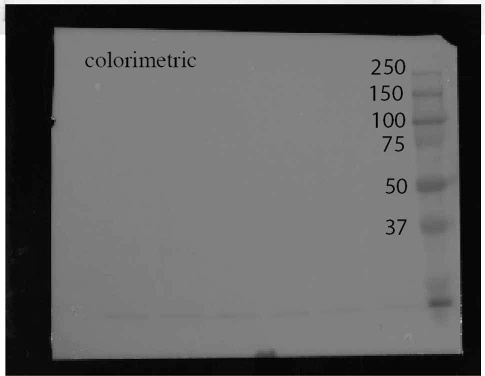

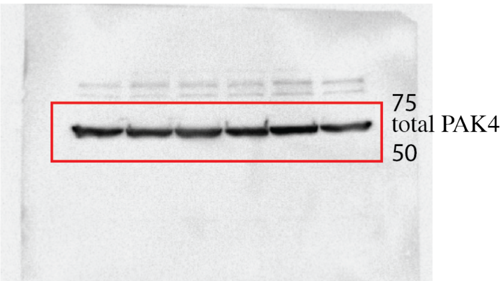

outlined in red belongs to panel 5D tPak4

captured with anti-rabbit HRP using Bio-Rad chemiluminescent system SuperSignal West Pico
