# Supplementary figures and images for: IODVA1, a guanidinobenzimidazole derivative, targets Rac activity and Ras-driven cancer models
Source: PLoS One. 2020 Mar 12;15(3):e0229801. doi: 10.1371/journal.pone.0229801 (PMC7067412; doi:10.1371/journal.pone.0229801)

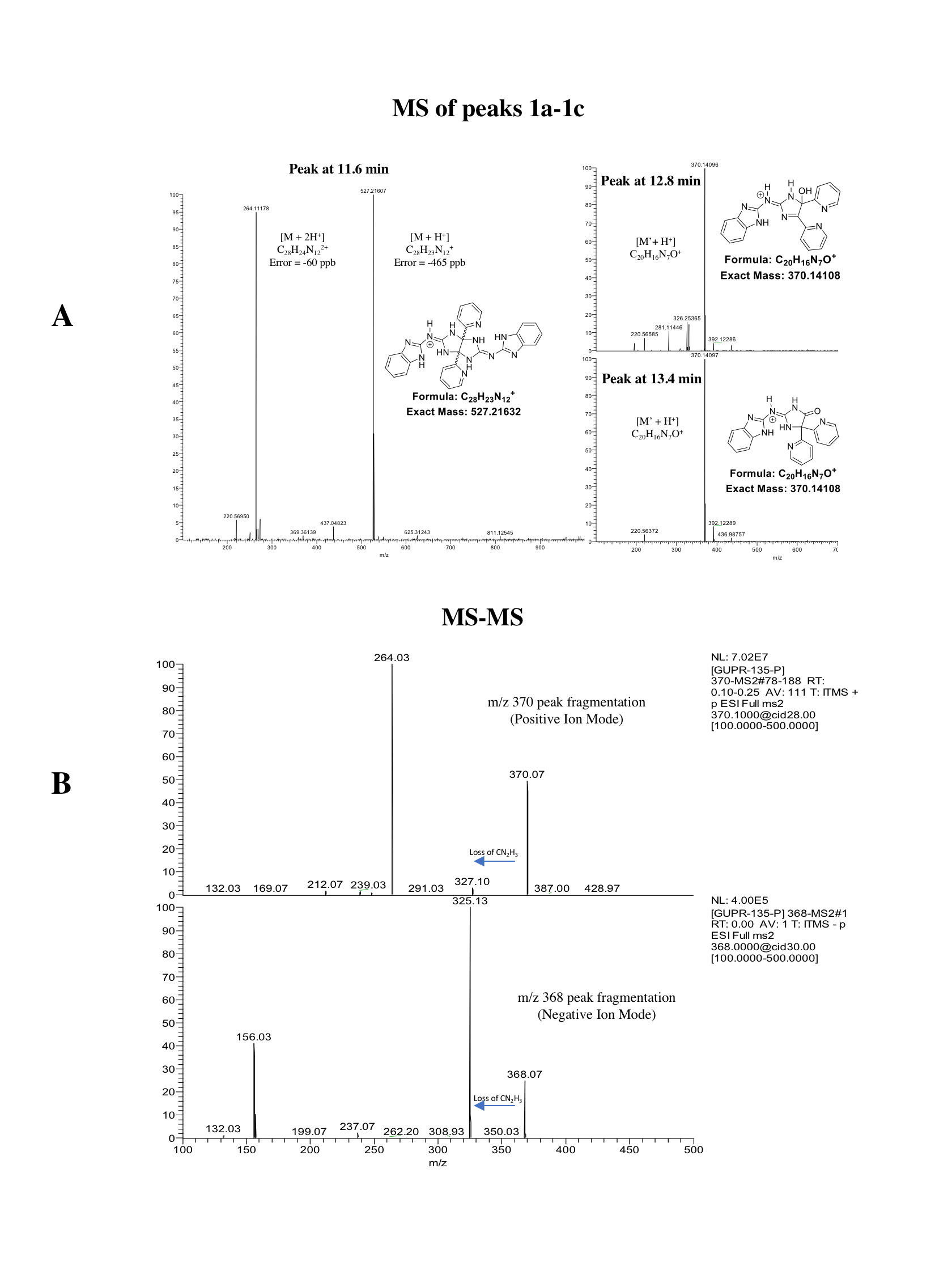

Supplement: S1 Fig — (A) Electrospray ionization spectrum of the 3 peaks at 11.6, 12.8, and 13.4 minutes, respectively. Structure of each component is shown. (B) MS-MS fragmentation of the 370.1409 peak of IODVA1. (TIF) [file pone.0229801.s002.tif]

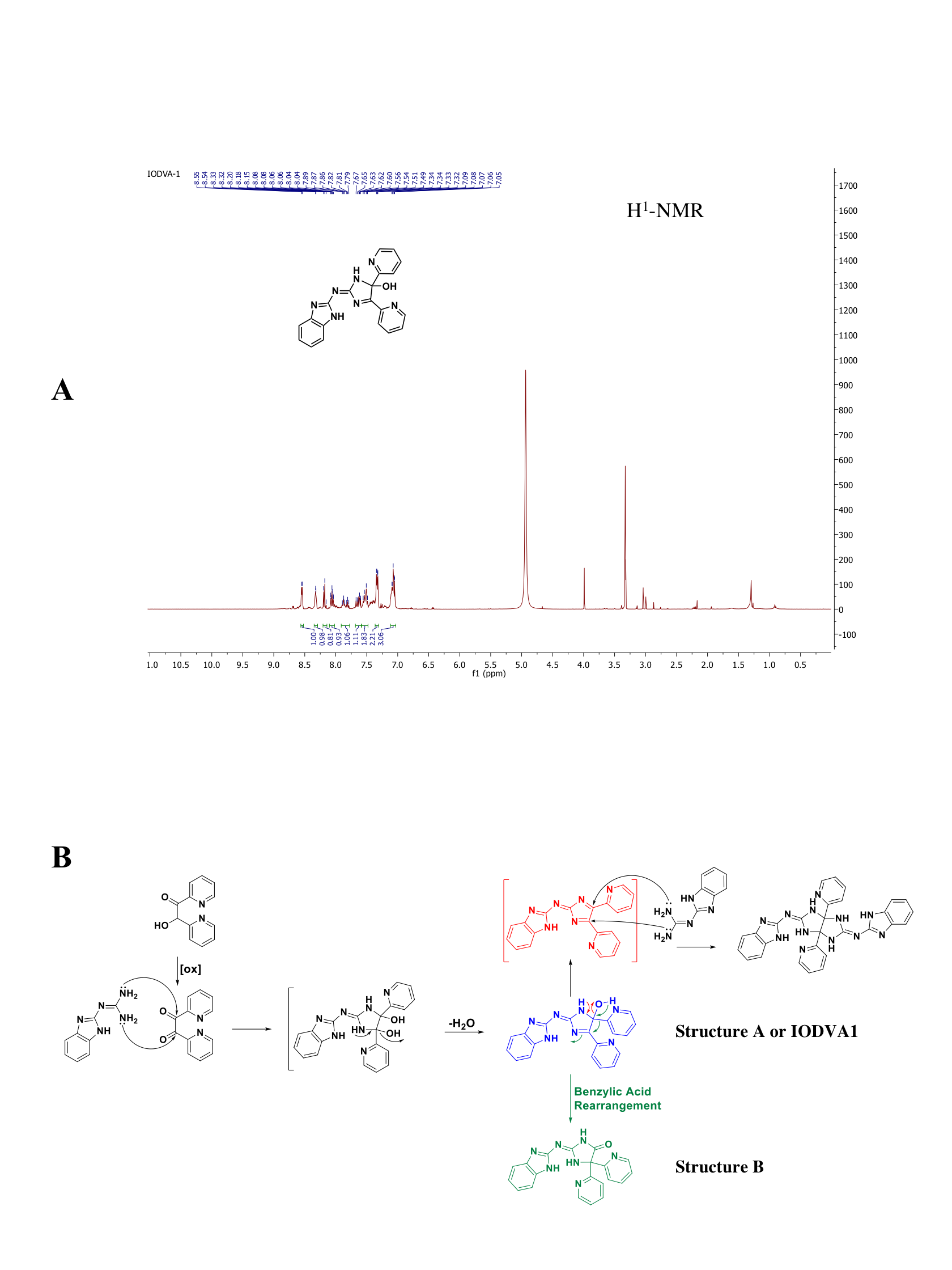

Supplement: S2 Fig — (A) 1H NMR of synthesized IODVA1 in methanol-d4. (B) Proposed mechanism of the reaction between 2-guanidinobenzimidazole and α-pyridoin resulting in structures A and B, NSC124205, and other products. (TIF) [file pone.0229801.s003.tif]

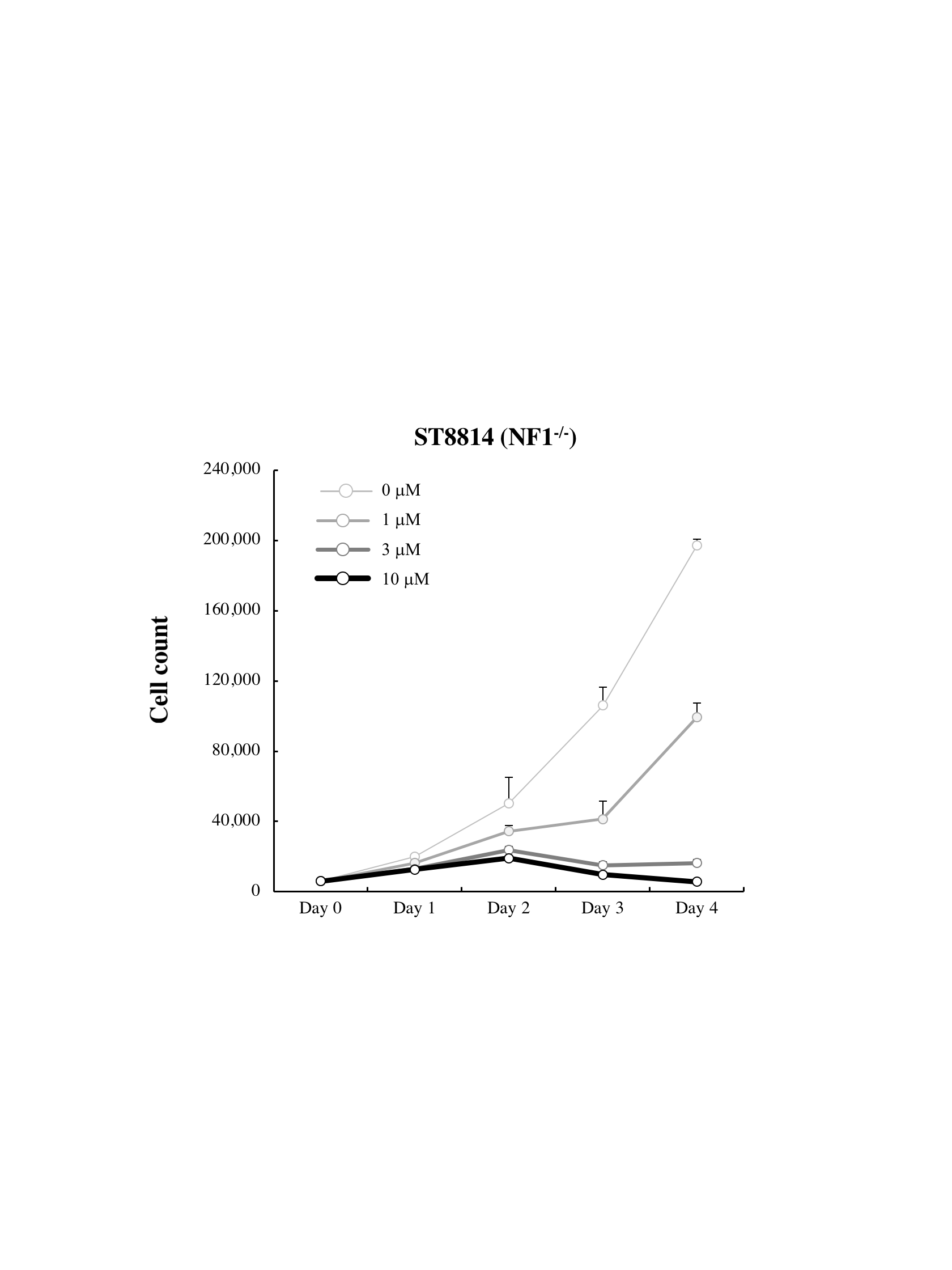

Supplement: S3 Fig — ST8814 cells were grown in the presence of the indicated IODVA1 concentrations and counted daily for 4 days. Each dot and bar is the mean and standard deviation respectively, of 3 independent experiments. (TIF) [file pone.0229801.s004.tif]

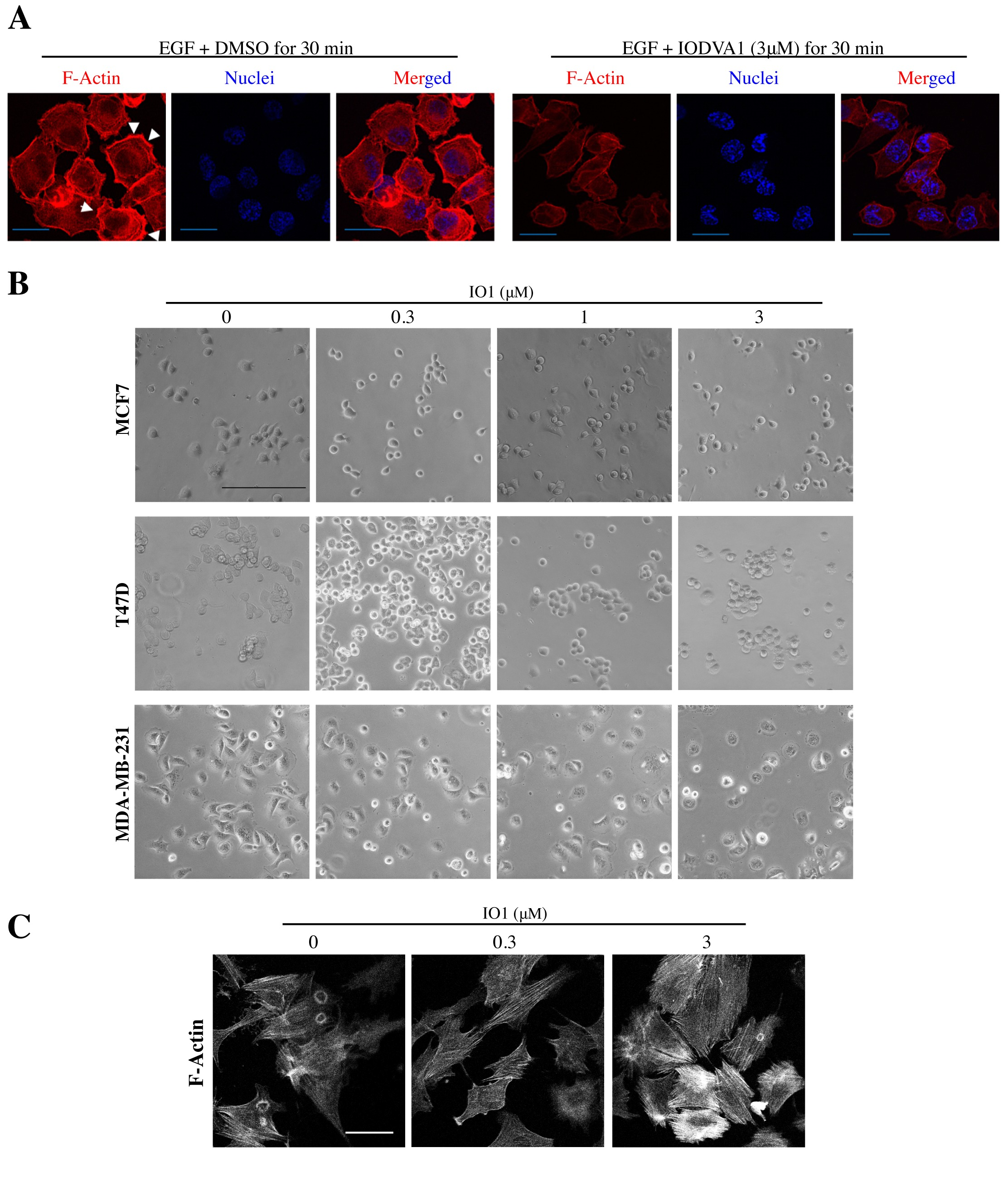

Supplement: S4 Fig — (A) MDA-MB-231 cells were EGF activated for 10 min, washed, treated with DMSO vehicle control or IODVA1 3 μM for 30 min, fixed, and stained for F-actin and nuclei (N = 3). Arrows point to lamellipodia structures. Images were taken at 100X. Scale bar = 10 μm. (B) Representative images from which main Fig 6A quantification was made. (C) Representative images of NIH-3T3 cells treated with the indicated concentrations of IODVA1 for 1 hours in serum-free media, fixed and stained with fluorescent phalloidin to visualize stress fibers. (TIF) [file pone.0229801.s005.tif]

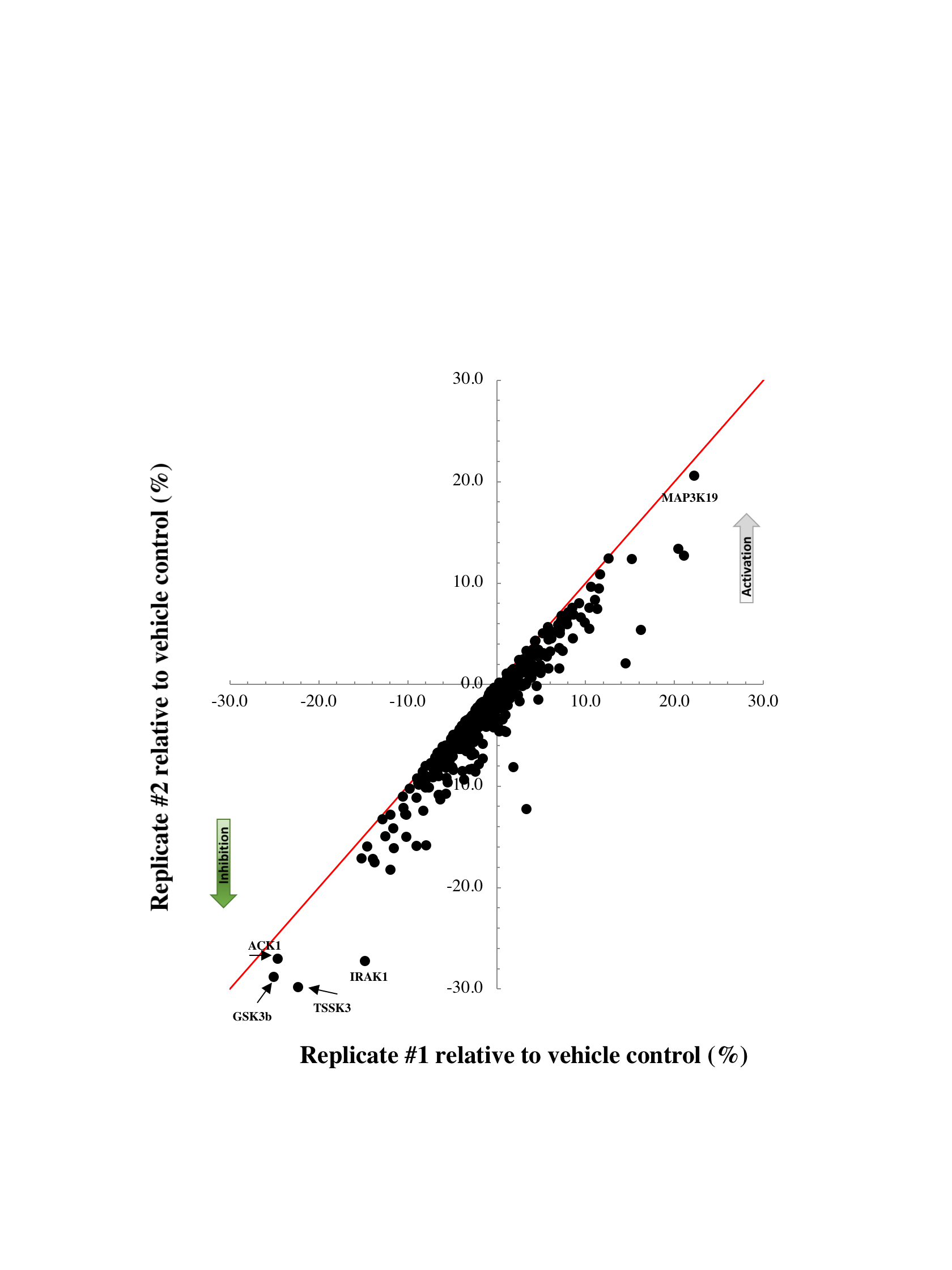

Supplement: S5 Fig — The activity of 369 kinases was tested twice in the presence of 0.5 μM IODVA1. Plotted is the remaining activity of replicate 1 vs 2 expressed as % of vehicle control set at 0% for each kinase. Kinases whose activities were decreased or increased by more than 3σ from mean are indicated. (TIF) [file pone.0229801.s006.tif]

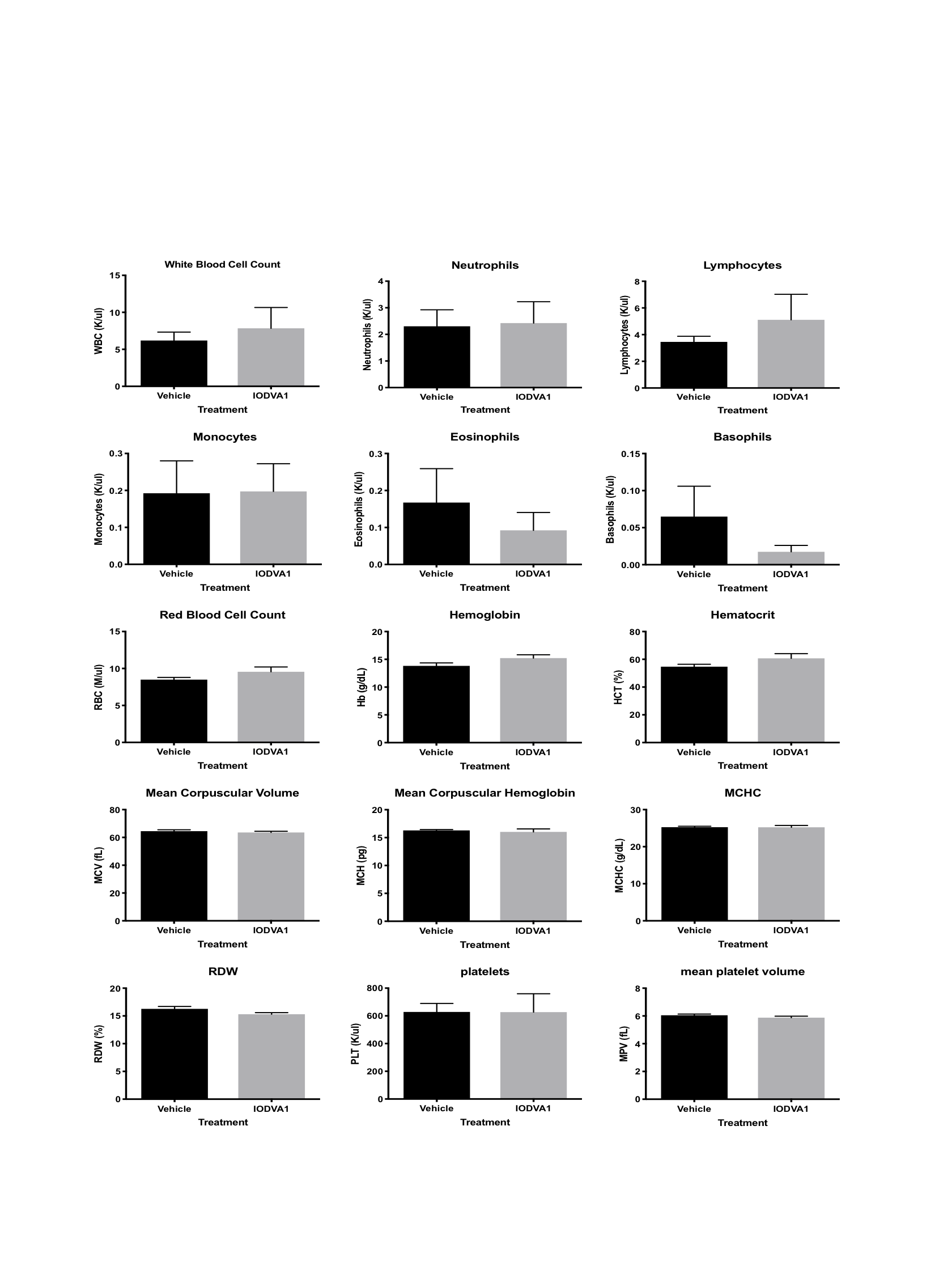

Supplement: S6 Fig — Peripheral blood collected after 12 doses of IODVA1 in tumor-bearing animals were analyzed for blood counts with a Hemavet. No statistically significant changes in blood counts were detected between vehicle control and IODVA1 treated animals (N = 4, mean ± stdev). (TIF) [file pone.0229801.s007.tif]
